# Report of a Case Verifying the Statement First Made by Dr. M. H. Cryer, Showing Direct Communication of the Frontal Sinus with the Antrum of Highmore

**Published:** 1897-12

**Authors:** T. W. Brophy

**Affiliations:** Chicago, Ill.


### Report of a Case Verifying the Statement First Made by Dr. M. H. Cryer, Showing Direct Communication of the Frontal Sinus with the Antrum of Highmore.

BY T. W. BROPHY, M.D., D.D.S., CHICAGO, ILL.

Abstract of a paper read at the American Dental Association, August, 1897.

The superior right bicuspid and molar teeth had been removed and a small opening made into the antrum before the case came into the hands of Dr. Brophy, irrigation being carried in by syringing daily with the usual antiseptic solutions. No satisfactory progress being made in arresting the disease, the patient was taken to Dr. B. for diagnosis. Exploration by means of a silver probe revealed no diseased bone, and a more extensive opening and also a more thorough drainage was recommended. The patient seemed at first to improve after the operation, but after home treatment for two or three months, returned in the original condition.

It was then decided to remove a greater portion of the anterior wall of the antrum. The operation revealed a thickening of the mucous membrane with polypi upon its surface, besides a small piece of rubber tubing which had been lost in the cavity. The cavity was thoroughly curetted, packed with boracic gauze, the packing was removed daily for about a week, when a hard rubber canula was made retaining the full size of the opening, preventing the closing of the opening by granulations and enabling to thorougly irrigate the cavity and watch the process of repair.

The accumulation of pus persisted at the upper nasal surface of the cavity, and after two or three months of the most vigorous treatment, it was decided that the frontal sinus must be involved. Accordingly an operation was made opening the sinus, which was found to be filled with pus. The anterior wall of the frontal sinus was removed exposing the cavity to view when it was found to be denuded of membrane. By carrying the incision downward along the inner canthus of the eye a silver probe was passed directly into the cavity of the antrum without obstruction. Water was forced into the frontal sinus which readily found its way through into the antral cavity. Pus also dribbled from the region of the ethmoid cells. These cells were curetted, some diseased bone removed, and the whole thoroughly irrigated. Irrigation and antiseptic dressings together with the insertion of a drainage tube, completed the operation, suppuration finally ceasing, though a thick glossy mucus occasionally comes from the nasal cavity. This case, in the judgment of the writer, had its origin in a dental alveolar abscess of the teeth which had been removed before the patient came to him. The cure of cases of this character could not possibly be effected by opening and treating the central cavity alone.

The feature especially presented was the anatomical relation of the frontal sinus, infundibulum and antrum of Highmore.

